# Visualization of gastric varices using angiographic C-arm CT during retrograde transvenous sclerotherapy

**DOI:** 10.4103/0971-3026.59751

**Published:** 2010-02

**Authors:** Jin Iwazawa, Shoichi Ohue, Hisashi Abe, Takashi Mitani

**Affiliations:** Department of Radiology, Nissay Hospital, 6-3-8 Itachibori, Nishiku, Osaka, Japan; 1 Department of Radiology, Komatsu Hospital, 11-6 Kawakatsucho, Neyagawa, Japan

**Keywords:** C-arm CT, gastric varix, sclerotherapy, venography

## Abstract

During retrograde transvenous sclerotherapy for gastric varices, sufficient opacification of the target varices on venography is essential for successful treatment. However, venography sometimes cannot identify target varices due to overlapping adjacent collateral vessels or leakage of contrast medium to other outflow veins. We report how C-arm CT images acquired using a flat-panel detector angiography system helped to identify target varices and predict the distribution of a sclerosant, which resulted in safer sclerotherapy and increased operator confidence.

## Introduction

Angiographic C-arm CT has recently been used for vascular intervention because it can acquire images of the vascular anatomy and provide information about the surrounding soft tissues.[1–5] The C-arm flat-panel angiography system provides multiplanar soft-tissue images as well as conventional planar angiograms from a single unit. Here, we assessed whether C-arm CT during retrograde transvenous sclerotherapy of gastric varices added additional useful information with regard to identification of gastric varices and prediction of the extent of sclerosant distribution in a single patient.

## Case Report

**Figure 1 (a-d):**
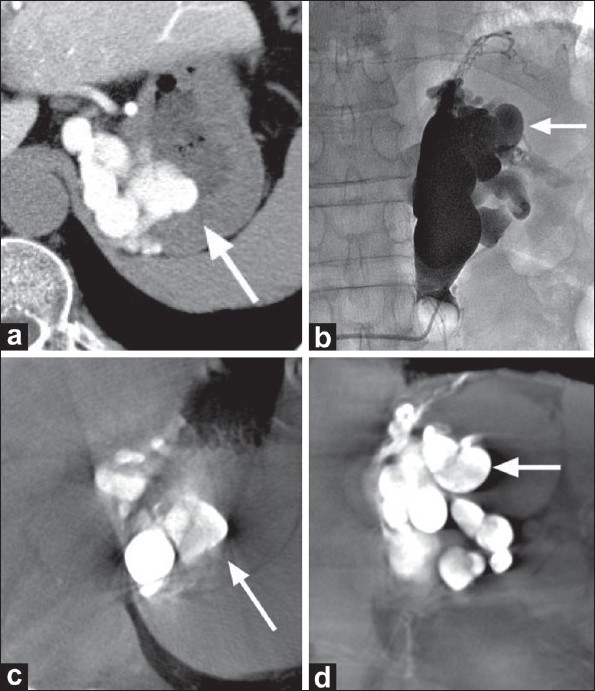
Comparative images during balloon-occluded retrograde transvenous sclerotherapy for gastric varices in a 64-yearold man with liver cirrhosis related to hepatitis C. Axial CT scans during arterial portography (a) and contrast-enhanced C-arm CT (c) both show gastric varices located at the gastric fornix with the same configuration (arrows). Frontal image of a balloon-occluded retrograde venogram from the left adrenal vein (b) delineates probable gastric varices (arrow) that were confirmed as target varices by referring to the corresponding contrast-enhanced C-arm CT coronal image (arrow) (d)

A 64-year-old man with liver cirrhosis related to hepatitis C was referred to our hospital with gastric varices. Contrast-enhanced multidetector CT (MDCT) showed gastric varices directly connected to a dilated left adrenal vein. Endoscopy revealed that the varices were located at the fornix of the stomach. First, we performed CT during arterial portography (CTAP) to assess the target varices and their inflow and outflow veins, using a 16-MDCT scanner (Somatom Sensation, Siemens Medical Solutions, Forchheim, Germany) with the following scanning parameters: 120 kV; 182 mAs; beam collimation, 0.75 mm; helical pitch, 1.15 mm; and rotation table speed, 0.5 s. Images were reconstructed in 5 mm-thick transverse sections to provide contiguous sections. A 4-Fr angiography catheter was placed in the proximal superior mesenteric artery before CTAP and through this iodinated contrast medium (90 ml) was delivered by a power injector at a rate of 3 ml/s. Image acquisition began at 25 s after starting contrast injection. This procedure revealed that the varices were approximately 3 cm in diameter and located at the fornix of the stomach [Figure 1a]. The major afferent veins were the left gastric vein, the posterior gastric vein, and the short gastric vein; the left adrenal vein was the only major drainage vessel. Because no other drainage veins were identified, retrograde transvenous sclerotherapy for the gastric varices was scheduled 35 days later; we planned to use a C-arm angiography system with a 30 × 30 cm flat-panel detector (Innova 3100, GE Healthcare, Waukesha, Wisconsin) set at the following parameters: Total scanning angle, 200°; rotation speed, 20°/s; matrix size, 1500 × 1500; isotropic voxel size, 0.2 mm; and effective field of view, 18 cm^2^. Raw data sets were transferred to an external workstation (Advantage Workstation 4.2, GE Healthcare), where images were reconstructed over a period of about 2.25 min to produce multisectional images. We positioned a 6.5-Fr balloon catheter (Artec Balloon Catheter, Create Medic, Yokohama, Japan) at the proximal left adrenal vein via the right femoral vein, and a left adrenal venogram was obtained by manually injecting contrast medium (300 mg of iodine; Iopamidol) while the balloon was inflated. Probable target varices were visualized on the venogram [Figure 1b]. To confirm that the probable varices were the actual targets, contrast-enhanced C-arm CT images were obtained by manually injecting the same volume of contrast medium (up to 100 mg of iodine) diluted with saline while the balloon was reinflated. The C-arm CT delineated the target gastric varices with the same configuration as the CTAP [Figure 1a and c]. Meanwhile, the probable varices visualized on conventional planar venogram were identified as the targets by referring to the corresponding C-arm CT coronal images [Figure 1b and d]. Because the varices were sufficiently opacified, 40 ml of 5% ethanolamine oleate in iodinated contrast medium was injected through the balloon catheter and the occlusion balloon was kept inflated for 1 h.

**Figure 2 (a-d):**
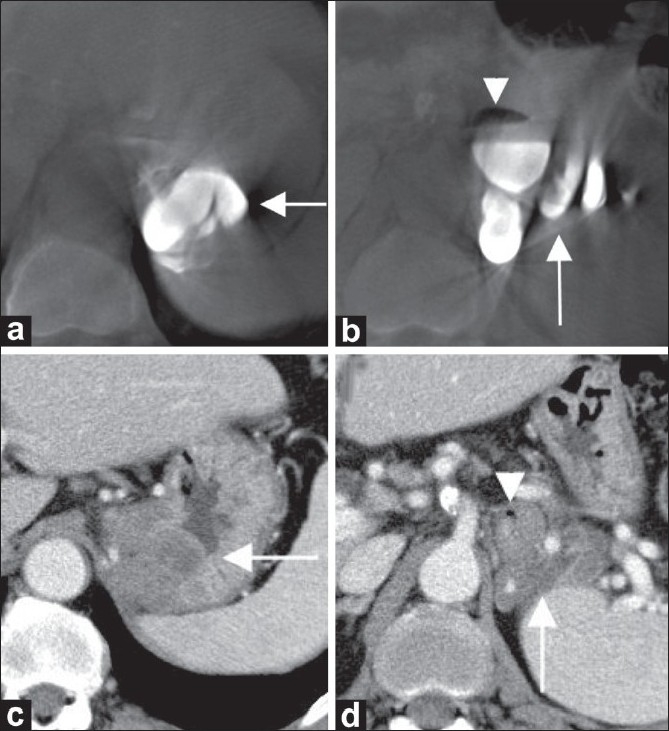
Unenhanced C-arm CT images (a,b) acquired just after the sclerosant injection and the corresponding contrast-enhanced MDCT images (c,d) obtained at 7 days after sclerotherapy to assess the sclerosant distribution. The transverse C-arm CT image (a) shows complete filling of the target varices (arrow) with sclerosant; this is confirmed by the corresponding axial CT scan (c), which demonstrates complete thrombosis of the target varices (arrow). The posterior gastric vein is entirely filled with the sclerosant (arrow in b), while the sclerosant is partially distributed to the gastrorenal shunt (arrowhead in b). Axial contrast-enhanced CT scan (d) also confirms that the posterior gastric vein is completely thrombosed (arrow), whereas the gastrorenal shunt remains patent (arrowhead). The sclerosant distribution seen in the unenhanced C-arm CT images coincides with that of the MDCT images

When the sclerosant was completely injected, unenhanced C-arm CT images immediately demonstrated total filling of the target varices with the sclerosant [Figure 2a] and partial filling of the gastrorenal shunt [Figure 2b]. Of the afferent gastric veins, sclerosant completely filled the posterior gastric vein [Figure 2b], but was not distributed to the left gastric vein. As much as possible of the sclerosant was manually aspirated. The balloon catheter was then carefully deflated and removed under fluoroscopic guidance. Contrast-enhanced MDCT was done 7 days after sclerotherapy for therapeutic evaluation. The distribution of the sclerosant visualized on unenhanced C-arm CT images obtained immediately after injection was comparable with that of the thrombosed gastric varices observed on post-therapeutic MDCT [Figure 2a and c]. The MDCT also confirmed that the posterior gastric vein was completely thrombosed [Figure 2b and d], whereas the left gastric vein remained patent.

## Discussion

Gastric varices with spontaneous gastrosystemic shunts have been recently treated by retrograde sclerotherapy through the outflow veins of the varices.[6–8] The outcome of treatment depends on proper visualization and identification of the intended target varices using balloon-occluded retrograde venography through the gastrosystemic shunt. Gastric varices can sometimes be partially overlapped by surrounding dilated collaterals or are not opacified due to leakage of contrast medium into the systemic circulation via other outflow vessels during conventional venography. Therefore, venography with different projections is needed to confirm whether target varices are sufficiently visible and opacified before treatment. C-arm angiography using a flat-panel detector is a novel imaging modality that can generate both conventional planar angiograms and low-contrast soft tissue images in multiple planes. C-arm CT for abdominal interventions has recently been clinically applied,[1–5,9] but its utility in balloon-occluded retrograde transvenous sclerotherapy for gastric varices has not been described.

In our patient we did C-arm CT, which provided additional information regarding the target varices, and this increased confidence among the angiography operators. Although the target varices of this patient were subsequently visualized by venography, the possibility that they might not be opacified due to excessive outflow into the gastrosystemic shunt could not be excluded using only venography. On the other hand, C-arm CT was useful for assessing the exact location and extent of opacification of target varices, because it constantly visualizes the intended target varices with surrounding soft tissue structures similar to MDCT. Furthermore, unenhanced C-arm CT obtained immediately after injecting the sclerosant accurately can predict the extent of sclerosant distribution. This technique allows additional sclerosant injection before the varices become thrombosed if the sclerosant distribution within the target varices is insufficient on post-therapeutic C-arm CT images. Moreover, post-therapeutic C-arm CT and MDCT were found to be comparable in their ability to evaluate thrombosis of varices and their related veins and therefore it may be possible to avoid additional contrast-enhanced MDCT to evaluate the outcome of sclerotherapy.

The C-arm CT imaging described herein has several limitations. Our C-arm angiography system was equipped with a flat-panel detector of 30 cm^2^ that produces an effective field of view of 18 cm^2^, which is quite limited and results in truncated images of the gastric veins or the gastrosystemic shunts. C-arm CT imaging requires longer setup and reconstruction times than conventional venography to produce images. Furthermore, the low-contrast nature of C-arm CT imaging requires administration of additional contrast medium for proper visualization of target varices and surrounding structures.

## Conclusion

C-arm CT provides substantially more information about the target varices (with regard to identification and opacification) than does conventional venography; Like MDCT, it can visualize the target varices with surrounding soft-tissue structures during transvenous sclerotherapy of gastric varices. C-arm CT is also useful for predicting sclerosant distribution within target varices and related veins during injection procedures, which allows additional sclerosant injection before the varices become thrombosed if the sclerosant is insufficiently distributed within the targets. C-arm CT allows operators to assess therapeutic outcomes with increased confidence.
